# Acetylcysteine increases sensitivity of ceftazidime-avibactam–resistant *enterobacterales* with different enzymatic resistance to ceftazidime-avibactam in vitro and in vivo

**DOI:** 10.1186/s12866-023-03068-5

**Published:** 2023-11-03

**Authors:** Zeyu Huang, Yijia Han, Xiaotuan Zhang, Yao Sun, Yuzhan Lin, Luozhu Feng, Tieli Zhou, Zhongyong Wang

**Affiliations:** 1 Department of Clinical Laboratory, Key Laboratory of Clinical Laboratory Diagnosis and Translational Research of Zhejiang Province, The First Affiliated Hospital of Wenzhou Medical University, Wenzhou, Zhejiang Province China; 2 Department of Medical Lab Science, School of Laboratory Medicine and Life Science, Wenzhou Medical University, Wenzhou, Zhejiang Province China

**Keywords:** *Enterobacterales*, Ceftazidime-avibactam, Antimicrobial resistance, Acetylcysteine

## Abstract

**Background:**

Ceftazidime-avibactam (CZA) improves treatment outcomes for infections caused by carbapenem-resistant organisms, but has led to serious bacterial resistance. Acetylcysteine (NAC) is an approved medication that protects the respiratory tract through antioxidant and anti-inflammatory effects.

**Results:**

This study found that NAC combined with CZA effectively inhibits the growth of CZA-resistant clinical *Enterobacterales* strains. The CZA/NAC combination inhibits biofilm formation in vitro and decreases bacterial burden in a mouse thigh infection model. The combination is biocompatible and primarily increases cell membrane permeability to cause bacterial death.

**Conclusions:**

These findings prove that the CZA/NAC combination has potential as a treatment for CZA-resistant *Enterobacterales* infections.

**Supplementary Information:**

The online version contains supplementary material available at 10.1186/s12866-023-03068-5.

## Background

 Antimicrobial resistance is a severe threat to human health, with an estimated 4.95 million fatalities linked to bacterial antimicrobial resistance in 2019 [1]. Carbapenem resistance is a major global public health threat, often resulting in severe morbidity and mortality [2, 3]. There is an urgent need for effective antimicrobial agents [4], and ceftazidime-avibactam (CZA) has shown promise in treating carbapenem-resistant infections [3, 5]. However, increasing bacterial resistance to CZA has been reported since its first use in clinics, mainly due to enzymatic resistance [3, 5–7]. CZA has been reported to inhibit the activities of Ambler class A, C, and some class D enzymes, but is ineffective against class B (including NDM-1, NDM-5, IMP) and some class D (OXA-23) enzymes [3]. Therefore, novel strategies are warranted to expand the spectrum of CZA and address concerns related to CZA resistance.

Acetylcysteine (NAC) is used to treat chronic bronchopulmonary diseases and acetaminophen overdose. NAC can be ingested orally, administered intravenously, or inhaled as a mist [8], and has gained attention owing to its antimicrobial activity [9]. Studies have demonstrated the antibacterial and biofilm-inhibitory effects of NAC against microorganisms in vitro [10, 11]. NAC has also shown synergistic effects with certain antibiotics against Pseudomonas aeruginosa [12, 13].

Therefore, it is intriguing to study the effect on NAC in combination with another agent. There are no reports investigating the effect of the combination of NAC and CZA against CZA-resistant bacterial strains. In this direction, the present study aimed to evaluate the synergistic activity of the CZA/NAC combination and offer novel potential treatment approaches to treat infections caused by CZA-resistant bacteria with different enzymatic resistance, including NDM-1, NDM-5, IMP, and OXA-23.

## Results

### The majority of CZA-resistant strains were found to exhibit multidrug resistance (MDR) phenotypes

The minimum inhibitory concentrations (MICs) of the tested antibiotics for all isolates (2 *Escherichia coli*, 4 *Enterobacter cloacae*, and 2 *Klebsiella pneumoniae*) are shown in Table S1. Most clinical isolates exhibited MDR characteristics. All strains were resistant to cefazolin, cefotetan, ceftriaxone, cefepime, and cotrimoxazole. There was no common agent that exhibited good antimicrobial effects on all CZA-resistant strains. Only amikacin showed potent antimicrobial activity against most tested isolates.

### The CZA/NAC combination exerted synergistic antimicrobial effects on most CZA-resistant strains with different resistance mechanisms

The strains displayed different enzymatic resistance mechanisms to CZA, including NDM-1, NDM-5, IMP, and OXA-23. The identification of β-lactamase genes and the characterization of other resistance elements could be found in our earlier research publications [14]. The synergism between CZA and NAC was evaluated using the checkerboard method. Table 1 demonstrates that the fractional inhibitory concentration index (FICI) values for the CZA/NAC combination were ≤ 0.5 for almost all tested strains, indicating significant synergism. The only exception was reported with FK7513 (FICI ≤ 0.75, additive effect or synergy). When used as a combination, NAC significantly reduced (≥ 2- to 8-fold decrease) the MIC of CZA. The combination of NAC and CZA did not exhibit any antagonistic effect in all tested strains.

**Table 1:** Mechanism of CZA resistance and antimicrobial susceptibility of the CZA/NAC combinations against the 8 clinical isolates used in this study

Strain	Antimicrobial resistance mechanism	Antimicrobial susceptibility (MIC, µg/mL)		Antimicrobial combination (MIC, µg/mL)			
		CZA	NAC	CZA + NAC	FICI	Potentiation	Interpretation
DC7914	*NDM-5*	≥ 512/4	≥ 1024	128/4 + 256	≤ 0.5	≥ 4-fold	synergy
DC8439	*NDM-1*	≥ 512/4	≥ 1024	128/4 + 256	≤ 0.5	≥ 4-fold	synergy
CG1090	*NDM-1*	≥ 512/4	≥ 1024	128/4 + 256	≤ 0.5	≥ 4-fold	synergy
CG1257	*IMP*	≥ 512/4	≥ 1024	64/4 + 128	≤ 0.25	≥ 8-fold	synergy
CG1381	*OXA-23*	≥ 512/4	≥ 1024	128/4 + 128	≤ 0.375	≥ 4-fold	synergy
CG1737	*NDM-5*	≥ 512/4	≥ 1024	128/4 + 128	≤ 0.375	≥ 4-fold	synergy
FK7018	*NDM-1*	≥ 512/4	≥ 1024	128/4 + 32	≤ 0.28125	≥ 4-fold	synergy
FK7513	*NDM-5*	≥ 512/4	≥ 1024	256/4 + 256	≤ 0.75	≥ 2-fold	additive effect/synergy

### The CZA/NAC combination demonstrated significant antibacterial activity in vitro

**Fig. 1:**
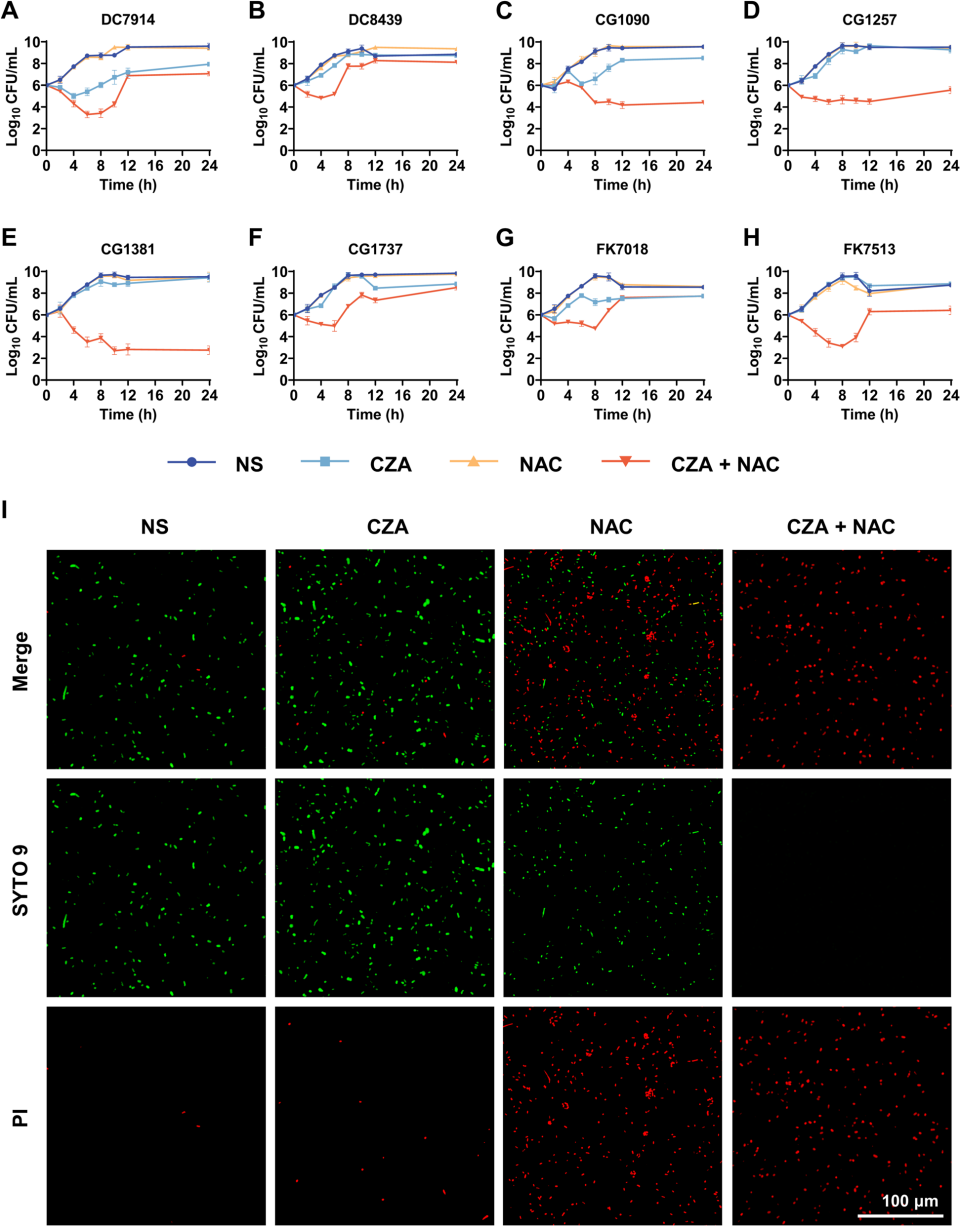
In vitro antibacterial activity of the CZA/NAC combination. Bactericidal kinetics against the CZA resistant **A**-**B** *Escherichia coli*, **C**-**F** *Enterobacter cloacae*, and **G**-**H** *Klebsiella pneumoniae*. **I** Fluorescence images of CZA–resistant Escherichia coli after incubation with natural saline (NS) and CZA/NAC combination, as observed in the live/dead staining assay

All strains were subjected to a time-kill assay to examine the effect of this combination on the growth kinetics of CZA-resistant strains. Based on the checkerboard analysis, a FICI value ≤ 0.75 was used to determine the drug concentration for the time-kill curve. As shown in Fig. 1A-H, the CZA/NAC combination was efficient in inhibiting the growth of the test isolates within the first 6 h, except for CG1090 that showed a significant decrease after 8 h, as compared to the monotherapy groups and natural saline (NS) group. Five out of eight strains (DC7914, DC8439, CG1737, FK7018, and FK7513) started to regrow after 8 h. CZA monotherapy also showed a slight inhibitory effect on several strains (DC7914, CG1090, FK7018). Overall, these results indicate that a minimum reduction of 2 log_10_ CFU/mL was observed during the first 6 h of the experiment for most bacterial cultures treated with the CZA/NAC combination. To further characterize the antibacterial activity of the CZA/NAC combination, a live/dead viability assay was performed. DC8439 was randomly selected as the experimental strain and the drug concentration was chosen as per the results of the checkerboard analysis. In this experiment, the dead bacteria were marked with propidium iodide (PI) that emits red fluorescence, while alive bacterial cells were stained with the green fluorescent probe SYTO 9. As shown in Fig. 1I, the red fluorescence intensity of the NS group was weaker than that of other groups, indicating that most bacteria were alive after 4 h of incubation at 37℃ without any drug treatment. The bacterial suspension treated with CZA showed a slightly higher red fluorescence than the NS group, which indicated that CZA showed a weak antibacterial activity against resistant strains. In contrast, the CZA/NAC combination treatment group showed stronger red fluorescence than other groups and no green fluorescence signal, suggesting that practically all the bacterial cells were dead. One interesting finding is that NAC monotherapy treatment could kill the majority of the bacteria in NS.

### The CZA/NAC combination inhibited the formation of biofilm

**Fig. 2:**
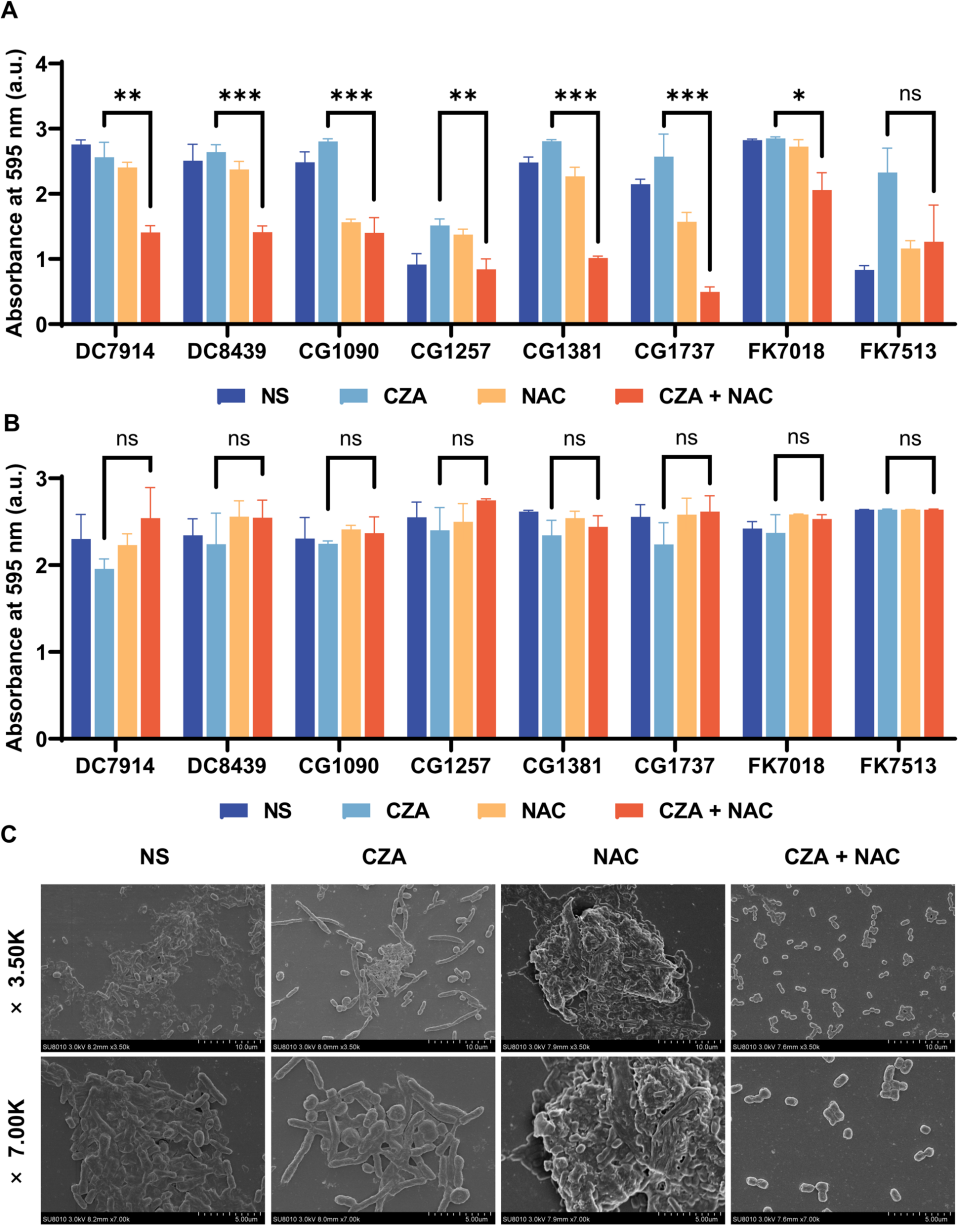
The antibiofilm activity of the CZA/NAC combination. **A** Biofilm-inhibitory effects of the CZA/NAC combination. **B** Biofilm disruption effects of the CZA/NAC combination. **C** The morphology of the biofilm after treatment with the CZA/NAC combination, as characterized by SEM

CZA and NAC were tested separately and together for their ability to inhibit the growth of biofilms and combat the already-formed biofilms by CZA-resistant strains. The drug concentration was determined based on the results of the checkerboard analysis. As shown in Fig. 2A, the CZA/NAC combination inhibited biofilm formation by all strains as compared to the CZA monotherapy. However, no considerable differences were observed between different treatment groups (Fig. 2B), which indicate that the CZA/NAC combination had no remarkable effect on combating mature biofilms. In order to further characterize the inhibitory effect of the CZA/NAC combination on biofilms, scanning electron microscopy (SEM) was performed. CG1381 was randomly selected as the experimental strain. As shown in Fig. 2C, the major field of vision was covered by the untreated bacterial cell biofilm in NS group. Biofilms were also observed in the groups treated with either CZA or NAC alone. However, the CZA/NAC combination could cause a significant decrease in the bacterial quantity and density. CZA treatment alone induced morphological changes in the bacterial cell structure, as evident from elongated cells, while NAC alone possibly caused cell membrane damage that resulted in bacterial cell clumping. These are some interesting observations reported in this experiment.

### The CZA/NAC combination effectively treated bacterial infection in vivo

**Fig. 3:**
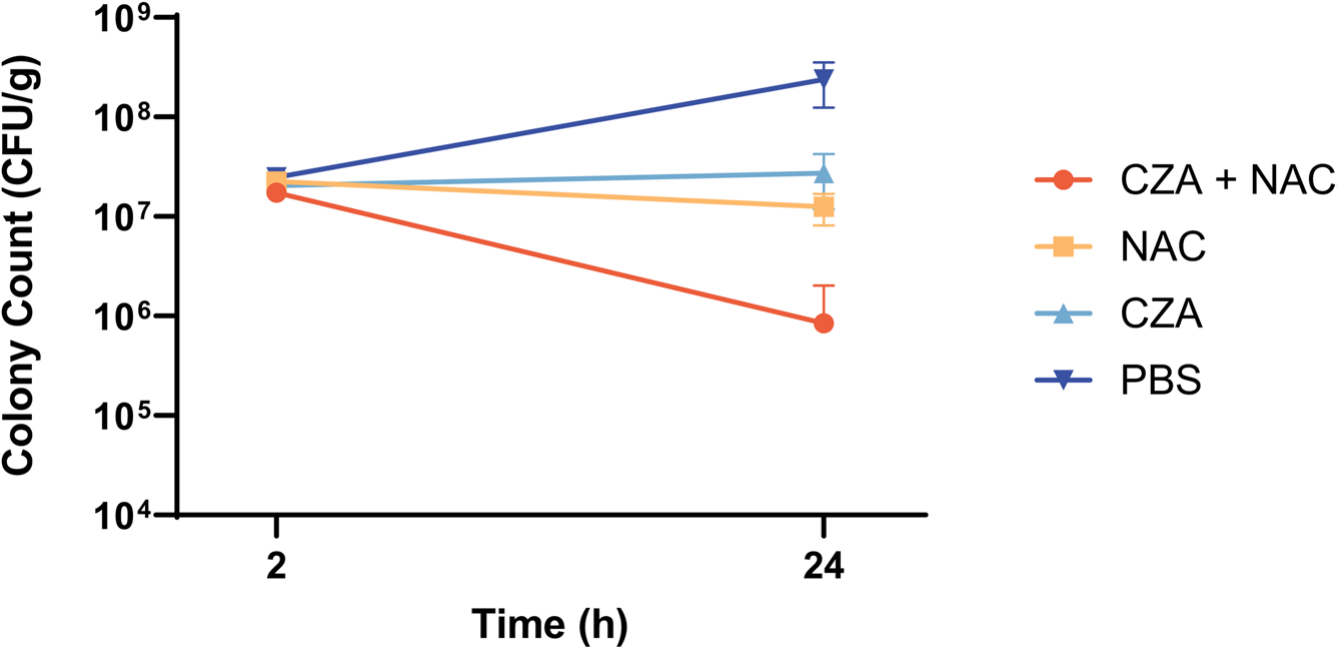
Quantified CFU/g in mouse thigh at 2 and 24 h

The effects of NAC (256 mg/kg) and CZA (128/32 mg/kg) were tested in a mouse thigh infection model. CG1381 was randomly selected as the experimental strain. As shown in Fig. 3, at the 24-hour time point, the CFU count in the mouse thigh tissues treated with PBS exhibited an approximate tenfold increase compared to the count at the 2-hour time point. However, the CFU counts in the mouse thigh tissues treated individually with NAC and CZA did not show significant changes. Importantly, it is noteworthy that the CFU count in the mouse thigh tissues subjected to combined NAC and CZA treatment at the 24-hour time point displayed an approximately one-tenth reduction in comparison to the count at the 2-hour time point.

### The CZA/NAC combination showed excellent biosafety in vitro and in vivo

**Fig. 4:**
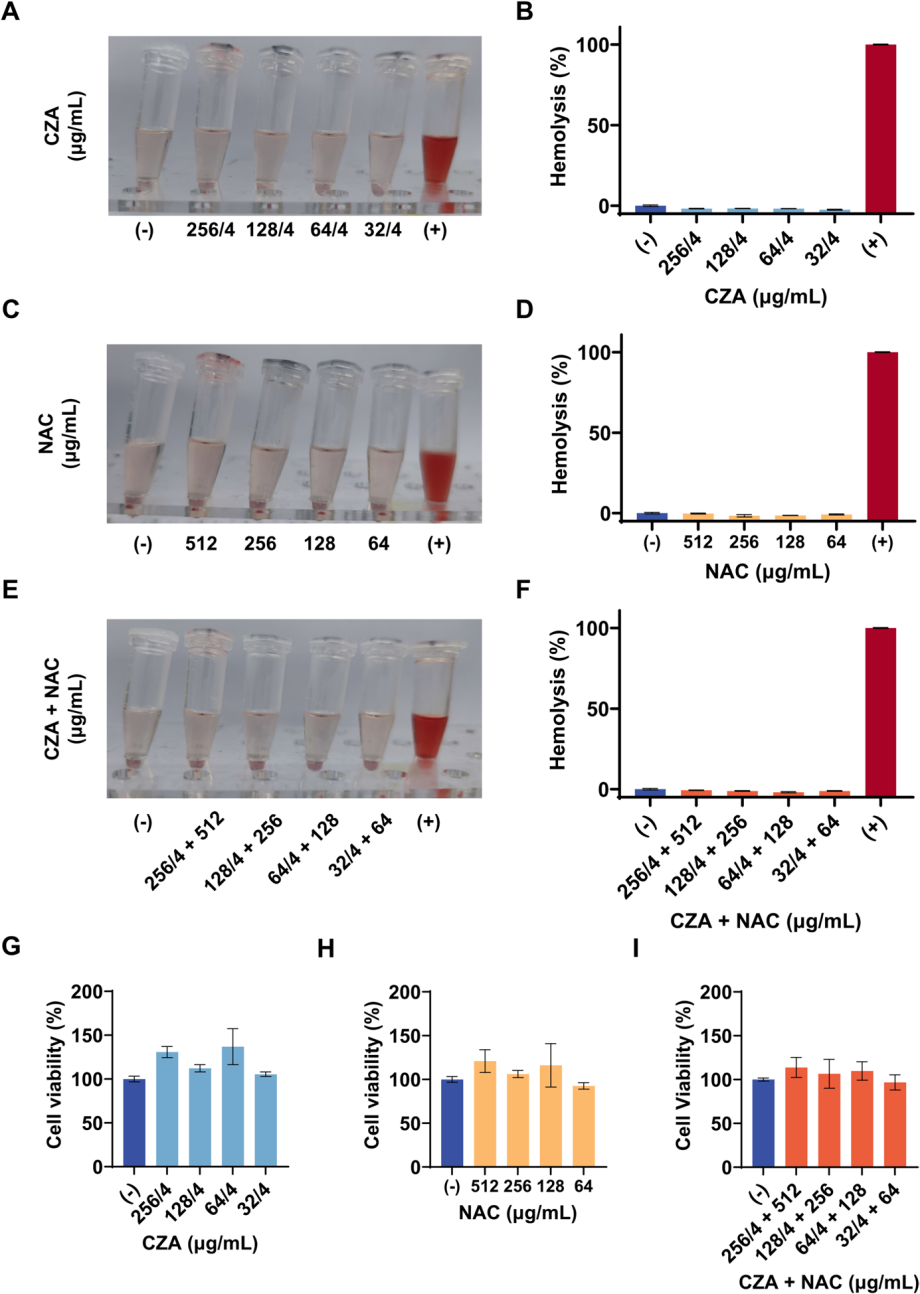
In vitro biocompatibility of the CZA/NAC combination. **A**-**F** Hemolysis analysis after treatment with various concentrations of the CZA/NAC combination. Erythrocytes incubated with PBS and 0.1% (by volume) Triton X-100 served as negative (−) and positive (+) control, respectively. **G**-**I** Viability of Huh-7 cells after treatment with the CZA/NAC combination

As biosafety is a critical consideration while designing novel antibacterial strategies, we performed hemolysis, cytotoxicity, and histopathology analyses to assess the biocompatibility of the CZA/NAC combination. As shown in Fig. 4A-F, the incubation of erythrocytes with various test agents did not result in obvious hemolysis. Figure 4G-I shows that there was no significant decrease in cell viability after treatment with different agents, indicating no additional toxicity to Huh-7 liver cancer cells. Furthermore, no obvious histological damage to major organs was observed (Figure S1A). As shown in Figure S1B-I, the main hematology indicators, including white blood cell, red blood cell, hemoglobin, hematocrit, mean corpuscular volume, mean corpuscular hemoglobin, and mean corpuscular hemoglobin, were all within the normal range. However, it should be noted that the monotherapy treatment led to a significant decrease in the level of platelets. The CZA/NAC combination treatment also led to a decrease in the amount of platelets, although no statistical difference was observed as compared to the PBS group. Thus, the clinical application of this treatment should be mediated with utmost care and attention.

### The CZA/NAC combination exerted a bactericidal effect mainly by increasing cell membrane permeability

**Fig. 5:**
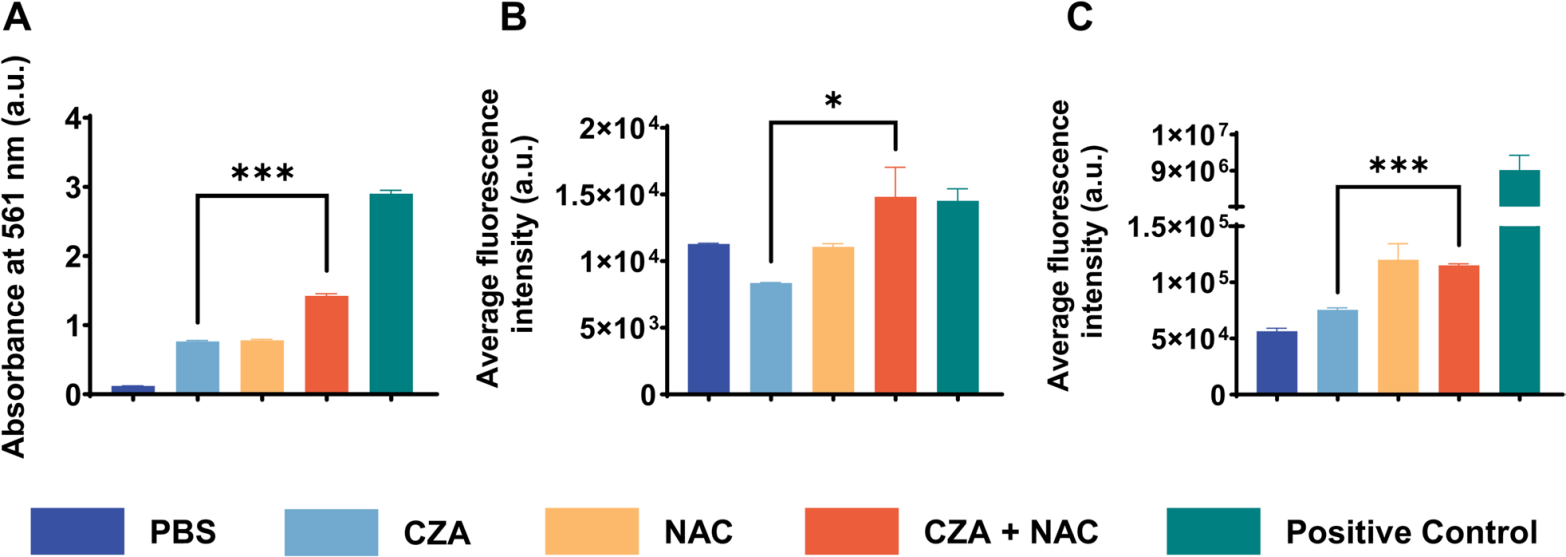
Antimicrobial mechanism of action of the CZA/NAC combination. **A** Intracellular protein leakage in CZA-resistant CG1381 after various treatments. **B** Membrane potential measurements of CZA-resistant CG1381 after various treatments. **C** ROS measurements of CZA-resistant CG1381 after various treatments

Considering that NAC could destroy the cell membrane and inhibit reactive oxygen species (ROS), it was reasonable to speculate that the synergistic antibacterial effect of NAC and CZA was mainly achieved through the damage caused to the bacterial cell membrane. To explore the underlying mechanism, we performed protein leakage, cell membrane permeability, and ROS detection assays. CG1381 was randomly selected as the experimental strain. As shown in Fig. 5A, three different treatment types were able to cause intracellular protein leakage. However, there was a noticeable increase in the intercellular protein leakage for the CZA/NAC combination group as compared to the CZA monotherapy treatment group. Figure 5B showed that the CZA/NAC combination treatment resulted in a noticeably higher fluorescence intensity than CZA monotherapy due to PI uptake and DNA binding, which proves the gradual deterioration of the cell membrane integrity. Surprisingly, as shown in Fig. 5C, NAC could substantially increase the ROS level, which might play a role in antibacterial activity. Considering that NAC is often used as a ROS inhibitor, but our research showed that NAC could promote ROS production, so we supplemented the strains for experiments (Figure S2). The results were still consistent with our original conclusion.

## Discussion

Since the first application of CZA in the clinical setting, there have been reports of microorganisms developing resistance to this agent [15–19]. Antimicrobial susceptibility tests revealed that most CZA-resistant isolates had MDR characteristics. The checkerboard method, time-kill curves, and live/dead bacterial cell viability assay showed that the CZA/NAC combination exhibited significant synergistic effects on most tested strains in vitro (except for FK7513, additive effect). As biofilm is a major cause of bacterial resistance to antibiotics and development of chronic infections [20, 21], we performed biofilm formation inhibition assay and SEM and found that the CZA/NAC combination could significantly reduce the formation of bacterial biofilms. We chose a mouse thigh infection model to evaluate the therapeutic effect of this combination treatment in vivo and observed that the CZA/NAC combination could significantly reduce bacteria in the mouse thigh tissue. In addition, hemolysis, cell viability, major blood cell parameters, and histopathology investigations demonstrated the high in vitro and in vivo biosafety of the CZA/NAC combination. We also detected intracellular proteins and nuclei from damaged bactericidal cells, and found that the CZA/NAC combination could significantly deteriorate the integrity of bacterial cell membrane.

Limitations of this study include: i) NAC monotherapy was effective in killing bacteria in NS but not in LB broth, possibly due to bacteria not growing in NS; ii) the mechanism of action and how NAC interacts with β-lactamases needs further exploration; iii) the safety, pharmacokinetics, and pharmacodynamics of long-term treatment in humans need evaluation; iv) further studies are needed to clarify why NAC, commonly used to inhibit ROS, increased ROS levels in CZA-resistant bacteria in this study; v) the crystal violet method employed in our study was primarily restricted to distinguishing between intact and disrupted biofilms, necessitating the utilization of more advanced techniques to differentiate between live and dead cells in the biofilm.

NAC can either increase or decrease bacterial sensitivity to antibiotics, and the interaction depends on several factors such as bacteria type, antibiotic type, and NAC concentration. Previous studies had shown that NAC acted synergistically with carbenicillin or ticarcillin to suppress *Pseudomonas aeruginosa* strains, but counteracted the effects of gentamicin and tobramycin [22]. Similarly, NAC could decrease the MIC of penicillin and ampicillin but increase the MIC of erythromycin and ciprofloxacin [23]. However, the exact mechanism of action and interaction between NAC and antibiotics are not clear and require further in vitro and in vivo studies. Besides, NAC is commonly used as a ROS inhibitor; however, our results demonstrate an increase in ROS levels following NAC treatment. This finding aligns with previous studies such as Li et al. [24], which also showed that NAC can increase bacterial membrane permeability and, as a result, ROS levels. Additionally, research by Petr Mlejnek et al. on human leukemia HL-60 and U937 cells suggested that NAC alone can elevate ROS levels [25]. Studies where NAC exhibits ROS-inhibitory effects are typically in comparison to situations where antimicrobial agents that inherently generate significant ROS are used alone. For example, Ana I Rodríguez-Rosado et al. found that ciprofloxacin alone generates substantial ROS, and when ciprofloxacin is used in combination with NAC, it significantly reduces ROS levels compared to ciprofloxacin alone [26]. The relationship between NAC and ROS is influenced by various factors including the target organism, dosage, duration of treatment, and other drug components. It’s a complex interplay that requires a case-by-case analysis.

## Conclusions

In conclusion, this is the first report of the synergistic efficacy of CZA in combination with NAC against CZA-resistant *Enterobacterales*. Our data demonstrates that the CZA/NAC combination has the potential for clinical application in the treatment of CZA-resistant bacterial infections.

## Methods

### Bacterial isolates

Eight non-duplicate clinical CZA-resistant *Enterobacterales* strains isolated from the First Affiliated Hospital of Wenzhou Medical University were selected. All isolates were identified using matrix-assisted laser desorption/ionization time-of-flight mass spectrometry (MALDI-TOF/MS; bioMérieux, Lyon, France). The VITEK 2 Compact System (bioMérieux, Lyon, France) was used to determine the MIC of the most common antibiotics, except for meropenem, which had its MIC assessed using the broth microdilution method. Briefly, An overnight-cultivated isolated bacterial colony was diluted to a McFarland standard of 0.5 in NS and then diluted once more to a 1:100 concentration in the cation-adjusted Mueller-Hinton broth (CAMHB). A series of two-fold dilutions of meropenem, spanning concentrations from 0.125 µg/mL to 256 µg/mL, was prepared. These dilutions were added to microplate wells along with the bacterial suspension. After 16 h of incubation at 37 °C, the MIC was identified as the lowest concentration of meropenem showing no visible growth. *Klebsiella pneumoniae* ATCC 700,603 was used as the quality control strain. The breakpoint score was interpreted using guidelines from the Clinical and Laboratory Standards Institute (CLSI, 2020).

### Checkerboard assay

CAMHB was used to dilute the two test agents at different concentrations according to MIC values for each isolate. An overnight-cultivated isolated bacterial colony was diluted to a McFarland standard of 0.5 in NS and then diluted once more to a 1:100 concentration in CAMHB. The ultimate bacterium concentration in each well was around 10^6^ CFU/mL. Results were reported after 16 h of incubation at 37℃. Three different tests were carried out. The synergistic effect of CZA and NAC was studied using the FICI value. FICI was calculated using the formula: FICI = FIC_A_ + FIC_B_; FIC_A_ = MIC_A_ in combination / MIC_A_ alone; FIC_B_ = MIC_B_ in combination / MIC_B_ alone. The interactions were explained as follows: FICI ≤ 0.5, synergistic effect; 0.5 < FICI ≤ 1, additive effect; 1 < FICI ≤ 2, irrelevant effect; antagonism of FICI > 2. Potentiation = MIC of CZA alone / MIC of CZA in combination.

### In vitro antibacterial activity

The concentration of NAC and CZA is consistent with the combined antimicrobial concentration in the checkerboard assay. For the bactericidal kinetic assay, bacteria (10^6^ CFU/mL) were treated with CZA and NAC separately or in combination. The bacterial suspensions were incubated at 37℃ with gentle shaking for 0, 2, 4, 6, 8, 10, 12, and 24 h. A previously prepared LB agar plate was evenly coated with 100 µL of diluent. The coated plates were incubated at 37℃ overnight for colony counting. For the live/dead bacterial cell viability assay, bacteria were treated with CZA and NAC at 37℃ for 4 h singly or in combination and the bacterial cells were stained with PI and SYTO 9 using the Live/Dead BacLight Bacterial Viability Kit, as per the manufacturer’s instructions. The samples were observed under a confocal microscope at excitation wavelengths of 488 and 561 nm and emission wavelengths of 530 nm (green) and 617 nm (red).

### Biofilm assay

The concentration of NAC and CZA is consistent with the combined antimicrobial concentration in the checkerboard assay. To investigate the effect of test agents on biofilm formation, bacterial suspension in LB medium (10^6^ CFU/mL) was added to a 96-well plate, then treated with CZA and NAC alone or in combination. After incubation for 24 h, the plate was washed, stained with 1% crystal violet. After staining for 15 min, the biofilms were washed and dried. To dissolve crystal violet, 200 µL of ethanol were added. The absorbance at 600 nm was measured by a microplate reader. Mature biofilm disruption assay was assayed similarly to biofilm formation assay. bacterial suspension in LB medium was added to a 96-well plate and grew overnight to form mature biofilm and then treated with CZA and NAC alone or in combination. After incubation for 24 h, the operation was the same as above. For SEM, silicon wafers were added with fresh bacterial suspension (10^6^ CFU/mL) and incubated with CZA and/or NAC. The treated samples were observed by SEM after fixation and dehydration.

### In vivo antibacterial activity

Male ICR mice (5-weeks-old) were obtained from Vital River, Beijing, China. Mice were maintained in accordance with the National Standards for Laboratory Animals of China. All animal researches were carried out in compliance with Wenzhou Laboratory Animal Welfare and Ethics standards. The posterior thigh muscle was injected with 100 µL of a bacterial suspension (10^8^ CFU/mL). After 2 h, the infected mice were randomly separated into four groups of six as follows: (i) PBS, (ii) CZA, (iii) NAC, (iv) CZA/NAC. Three mice from each group were euthanized immediately to collect the thigh tissues for bacterial load analysis. The harvested tissues were homogenized and subjected to serial dilution, followed by plating on LB agar plates to determine CFUs. Then, all mice from (ii), (iii), and (iv) groups were injected with CZA (128/32 mg/kg) and/or NAC (256 mg/kg) into the abdominal cavity. The injection volume was 200 µL. Mice from (i) group were injected with 200 µL of PBS. After 24 h, mice were euthanized, and infected thigh tissues of three mice from each group were collected. After being homogenized and serially diluted, the infected tissues were plated on LB agar plates for CFU titers.

### In vitro biocompatibility

For the hemolysis assay, mouse erythrocytes (3%) were incubated with different concentrations of CZA and NAC solutions alone or in combination for 2 h at 37℃. The samples were then centrifuged for 10 min at 4000 rpm. PBS was used as negative control, and 0.1% Triton X-100 was used as positive control. Hemolysis percentage (%) = (absorbance of sample − absorbance of negative control) / (absorbance of positive control − absorbance of negative control) × 100. The percentage of hemolysis was calculated by measuring the absorbance at 540 nm wavelength. For cell viability analysis, Huh-7 cells (2000 cells / well, 100 µL) were treated with CZA and NAC alone or in combination for 12 h. Later, 10 µL CCK-8 reagent was added to each well and the plate was incubated at 37 ℃ in the dark for 1 h. The absorbance at 450 nm was measured using a microplate reader. The percentage of cell viability was calculated as follows: Cell viability (%) = (absorbance of sample − absorbance of medium) / (absorbance of negative control − absorbance of medium) × 100.

### In vivo biocompatibility

Healthy mice were randomly divided into four groups of three as follows: (i) PBS, (ii) CZA, (iii) NAC, (iv) CZA/NAC. Mice from (ii), (iii), and (iv) groups were injected with CZA (128/32 mg/kg) and/or NAC (256 mg/kg) into the abdominal cavity. Group (i) were injected with PBS as a control. The injection volume was 200 µL. After 12 h, mice were injected with the same doses and euthanized after another 12 h. Major organs (heart, liver, spleen, lung, and kidney) were collected and fixed in a 4% formaldehyde solution. The tissues were paraffin-embedded and sliced into 5 μm sections. Subsequently, the organs were stained with H&E. The blood was obtained from each eye socket and used for routine blood tests. An animal-specific hematology analyzer was used for blood analysis.

### Study of antimicrobial mechanism

To investigate intracellular protein leakage, bacteria suspensions (10^8^ CFU/mL) were treated with CZA (128/4 µg/mL) and NAC (256 µg/mL) in PBS, alone or in combination. Bacteria suspensions treated with colistin (64 µg/mL, a well-known antimicrobial agent that is known to significantly disrupt bacterial cell membranes) were set as positive control.After being incubated at 37℃ for 4 h, bacterial suspensions were centrifuged at 5000 rpm for 10 min at 4℃. The BCA protein detection kit was used to quantify intracellular protein leakage in supernatants, as per the manufacturer’s instructions. The cell membrane permeability assay was performed by mixing bacterial suspensions (10^7^ CFU/mL) with CZA (128/4 µg/mL) and NAC (256 µg/mL) in PBS either alone or in combination. Bacteria suspensions treated with colistin (64 µg/mL) were set as positive control. After being incubated for 2 h at 37℃, PI was added at a final concentration of 50 µg/mL. The samples were scanned by a multifunctional microplate reader (BioTek Synergy NEO_2_, America) after 10 min at an excitation and emission wavelengths of 561 and 617 nm, respectively. For ROS detection, 500 µL of bacterial suspensions (10^6^ CFU/mL) were incubated with 10 µM dichloro-dihydro-fluorescein diacetate (DCFH-DA) at 37℃ for 45 min. DCFH-DA-loaded cells were then treated with CZA (128/4 µg/mL) and/or NAC (256 µg/mL) for 2 h at 37℃. Sterile distilled water was set as a blank control. DCFH-DA-loaded cells treated with Rosup (1:1000 diluted with PBS, provided by the assay kit) for 30 min at 37℃ were set as positive control. All samples were scanned using a multifunctional microplate reader (BioTek Synergy NEO_2_, America) at excitation and emission wavelengths of 488 and 535 nm, respectively.

### Statistical analysis

Data were expressed as mean ± standard deviation of at least three independent experiments. Statistical significance was determined using an independent two-tailed t-test and expressed as *P*-values < 0.05 (noted with*), 0.01 (noted with**), and 0.001 (noted with***). GraphPad Prism 9.4 was used for the statistical analysis.

### Supplementary Information

**Additional file 1:** **Figure S1.** *In vivo* biocompatibility of the CZA/NAC combination. (A) H&E staining images of main organs from mice after 24 h of various treatments. (B-I) Major blood cell parameters of mice after 24 h of various treatments. WBC, white blood cell; RBC, red blood cell; HGB, hemoglobin; HCT, hematocrit; MCV, mean corpuscular volume; MCH, mean corpuscular hemoglobin; MCHC, mean corpuscular hemoglobin concentration; PLT, platelet.

**Additional file 2:** **Figure S2. **ROS measurements of CZA-resistant DC7914, CG1090, and FK7018 after various treatments.

**Additional file 3:** **Table S1.** The MIC values against the 8 clinical isolates used in this study.

## Data Availability

All data generated or analysed during this study are included in this manuscript and its supplementary information files. The datasets used and analysed during the current study available from the corresponding author on reasonable request.

## Abbreviations

CZA: Ceftazidime-avibactam
NAC: Acetylcysteine
MDR: Multidrug resistance
MIC: Minimum inhibitory concentration
FICI: Fractional inhibitory concentration index
NS: Natural saline
PI: Propidium iodide
SEM: Scanning electron microscopy
H&E: Hematoxylin-eosin
PBS: Phosphate-buffered saline
ROS: Reactive oxygen species
MALDI-TOF/MS: Matrix-assisted laser desorption/ionization time-of-flight mass spectrometry
CAMHB: Cation-adjusted Mueller-Hinton broth
DCFH-DA: Dichloro-dihydro-fluorescein diacetate
