# Supplementary material for: Acetylcysteine increases sensitivity of ceftazidime-avibactam–resistant enterobacterales with different enzymatic resistance to ceftazidime-avibactam in vitro and in vivo
Source: BMC Microbiol. 2023 Nov 3;23:321. doi: 10.1186/s12866-023-03068-5 (PMC10623744; doi:10.1186/s12866-023-03068-5)
Supplement: Supplementary file 1 — Additional file 1: Figure S1. In vivo biocompatibility of the CZA/NAC combination. (A) H&E staining images of main organs from mice after 24 h of various treatments. (B-I) Major blood cell parameters of mice after 24 h of various treatments. WBC, white blood cell; RBC, red blood cell; HGB, hemoglobin; HCT, hematocrit; MCV, mean corpuscular volume; MCH, mean corpuscular hemoglobin; MCHC, mean corpuscular hemoglobin concentration; PLT, platelet. [file 12866_2023_3068_MOESM1_ESM.docx]

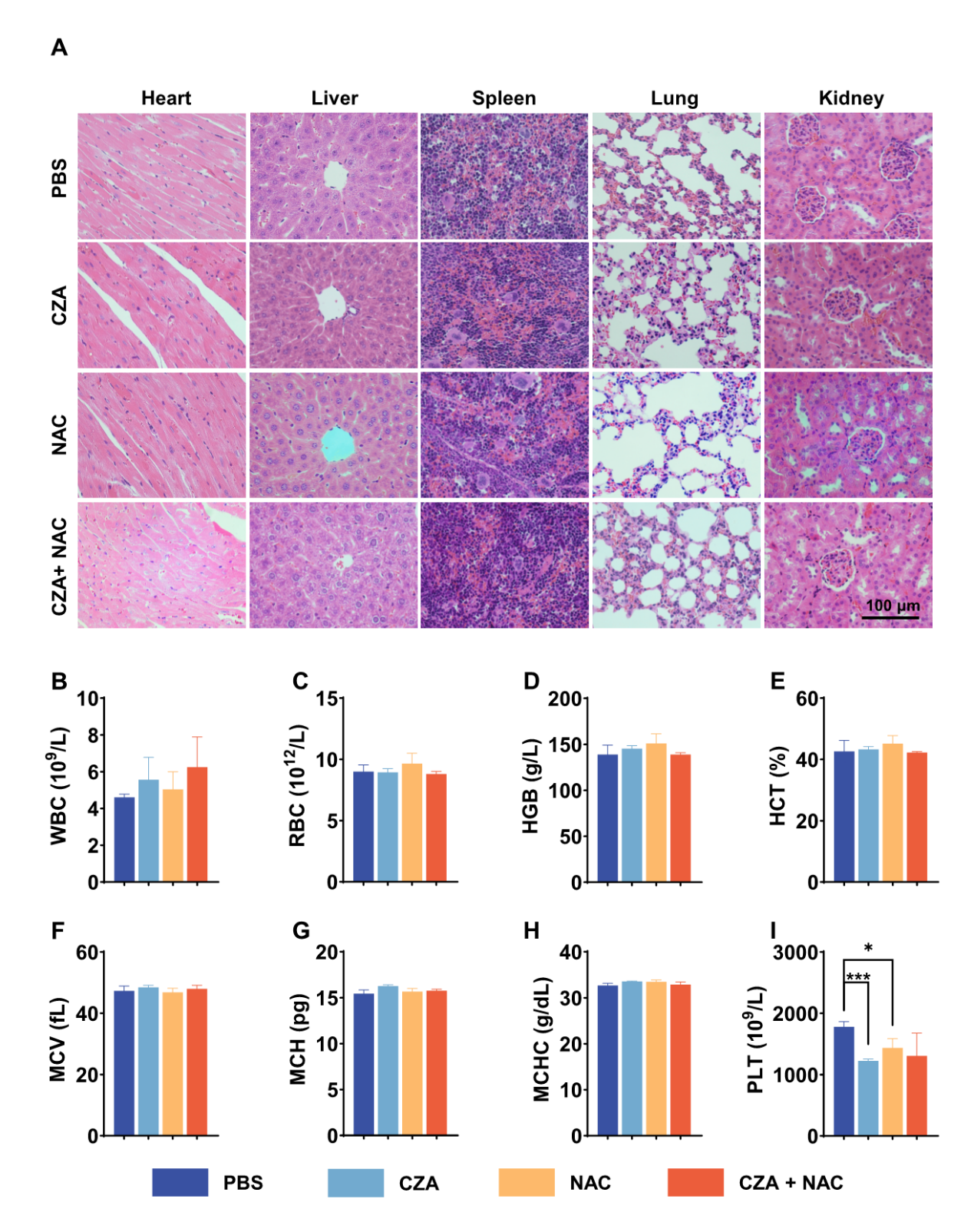


**Figure S1** *In vivo* biocompatibility of the CZA/NAC combination. (A) H&E staining images of main organs from mice after 24 h of various treatments. (B-I) Major blood cell parameters of mice after 24 h of various treatments. WBC, white blood cell; RBC, red blood cell; HGB, hemoglobin; HCT, hematocrit; MCV, mean corpuscular volume; MCH, mean corpuscular hemoglobin; MCHC, mean corpuscular hemoglobin concentration; PLT, platelet.
