# Supplementary figures and images for: Acetylcysteine increases sensitivity of ceftazidime-avibactam–resistant enterobacterales with different enzymatic resistance to ceftazidime-avibactam in vitro and in vivo
Source: BMC Microbiol. 2023 Nov 3;23:321. doi: 10.1186/s12866-023-03068-5 (PMC10623744; doi:10.1186/s12866-023-03068-5)

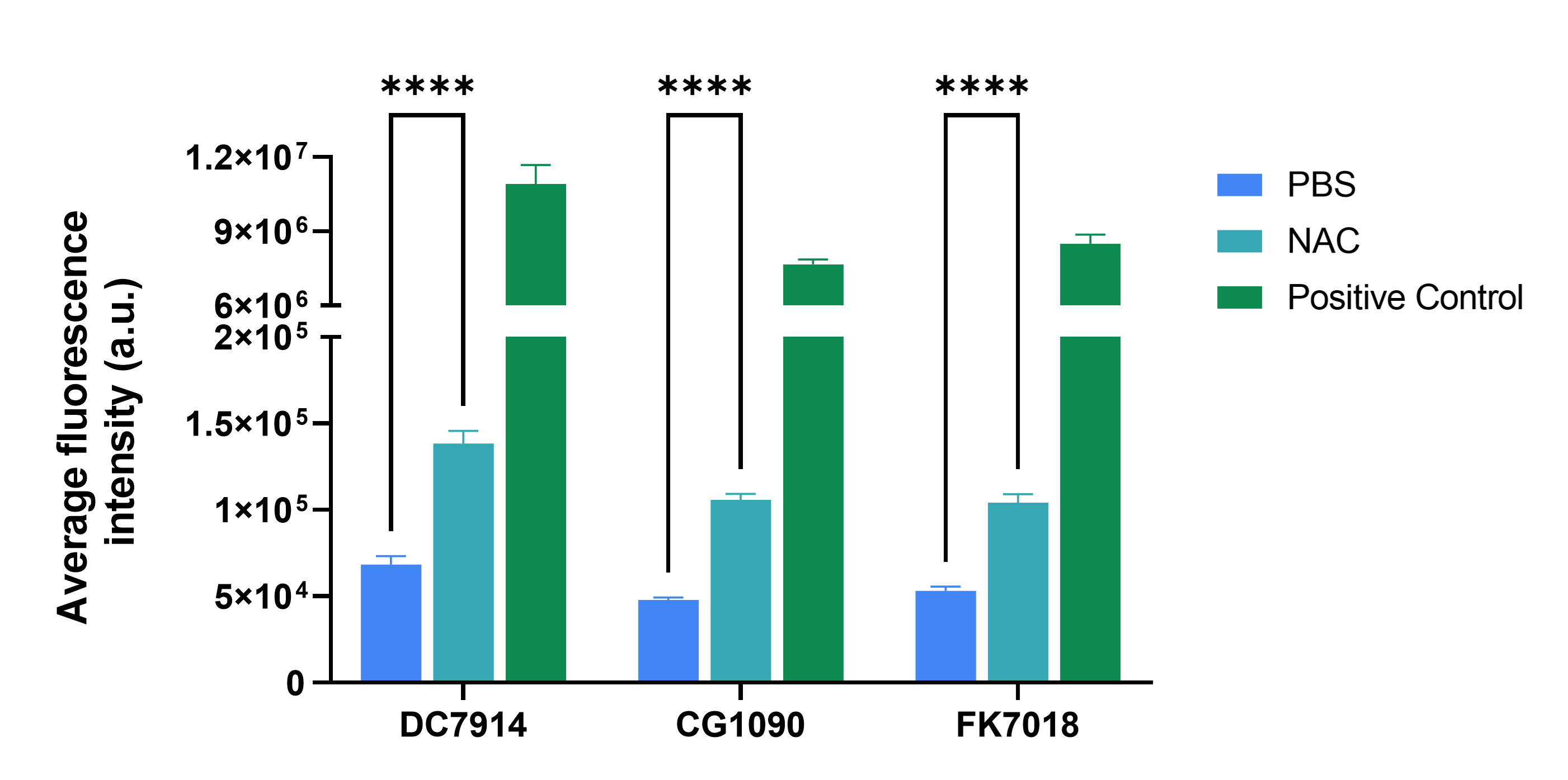


**Figure S2** ROS measurements of CZA-resistant DC7914, CG1090, and FK7018 after various treatments.

Supplement: Supplementary file 2 — Additional file 2: Figure S2. ROS measurements of CZA-resistant DC7914, CG1090, and FK7018 after various treatments. [file 12866_2023_3068_MOESM2_ESM.docx]
