# Supplementary material for: Acetylcysteine increases sensitivity of ceftazidime-avibactam–resistant enterobacterales with different enzymatic resistance to ceftazidime-avibactam in vitro and in vivo
Source: BMC Microbiol. 2023 Nov 3;23:321. doi: 10.1186/s12866-023-03068-5 (PMC10623744; doi:10.1186/s12866-023-03068-5)
Supplement: Supplementary file 3 — Additional file 3: Table S1. The MIC values against the 8 clinical isolates used in this study. [file 12866_2023_3068_MOESM3_ESM.docx]

| **Species** | **Strains** | **MIC values (μg/ml)** | | | | | | | | | | | |
| --- | --- | --- | --- | --- | --- | --- | --- | --- | --- | --- | --- | --- | --- |
|  |  | **CFZ** | **CTT** | **CRO** | **FEP** | **CIP** | **LVX** | **GEN** | **TOB** | **AMK** | **CTX** | **NIT** | **MEM** |
| ***E. coli*** | DC7914 | ≥64^R^ | ≥64^R^ | ≥64^R^ | ≥64^R^ | ≥4^R^ | ≥8^R^ | ≥16^R^ | ≥16^R^ | ≥64^R^ | ≥320^R^ | 64^I^ | 128^R^ |
|  | DC8439 | ≥64^R^ | ≥64^R^ | ≥64^R^ | ≥64^R^ | ≥4^R^ | ≥8^R^ | ≥16^R^ | 8^I^ | ≤2^S^ | ≥320^R^ | ≤16^S^ | 8^R^ |
| ***E. cloacae*** | CG1090 | ≥64^R^ | ≥64^R^ | ≥64^R^ | ≥64^R^ | ≥4^R^ | ≥8^R^ | ≥16^R^ | 8^I^ | ≤2^S^ | ≥320^R^ | 64^I^ | 8^R^ |
|  | CG1257 | ≥64^R^ | ≥64^R^ | ≥64^R^ | ≥64^R^ | ≤0.25^S^ | 1^S^ | 4^S^ | 8^I^ | ≤2^S^ | ≥320^R^ | 64^I^ | 4^R^ |
|  | CG1381 | ≥64^R^ | ≥64^R^ | ≥64^R^ | ≥64^R^ | 0.5^S^ | 1^S^ | 4^S^ | 8^I^ | ≤2^S^ | ≥320^R^ | 32^S^ | 32^R^ |
|  | CG1737 | ≥64^R^ | ≥64^R^ | ≥64^R^ | ≥64^R^ | ≥4^R^ | ≥8^R^ | 8^I^ | ≥16^R^ | ≤2^S^ | ≥320^R^ | 64^I^ | 64^R^ |
| ***K. pneumoniae*** | FK7018 | ≥64^R^ | ≥64^R^ | ≥64^R^ | ≥64^R^ | ≥4^R^ | 4^R^ | ≥16^R^ | 8^I^ | ≤2^S^ | ≥320^R^ | 128^R^ | 32^R^ |
|  | FK7513 | ≥64^R^ | ≥64^R^ | ≥64^R^ | ≥64^R^ | ≤0.25^S^ | ≤0.25^S^ | ≥16^R^ | ≥16^R^ | ≥64^R^ | ≥320^R^ | 128^R^ | 64^R^ |

**Table S1** The MIC values against the 8 clinical isolates used in this study.

**Abbreviations:**

CFZ, cefazolin; CTT, cefotetan; CRO, ceftriaxone; FEP, cefepime; CIP, ciprofloxacin; LVX, levofloxacin; GEN, gentamicin; TOB, tobramycin; AMK, amikacin; CTX, cotrimoxazole; NIT, nitrofurantoin; MEM, meropenem.

S, susceptible; I, intermediate; R, resistance.
